# Evolving Landscape of Paediatric Pneumococcal Meningitis in Argentina (2013–2023)

**DOI:** 10.3390/microorganisms13061301

**Published:** 2025-06-03

**Authors:** Jonathan Zintgraff, Paula Gagetti, Nahuel Sanchez Eluchans, Paulina Marchetti, María Alicia Moscoloni, Argentina Spn Working Group, Claudia Sara Lara, Alejandra Corso

**Affiliations:** 1 Servicio Bacteriología Clínica, Departamento de Bacteriología, Instituto Nacional de Enfermedades Infecciosas (INEI)—ANLIS “Dr. Carlos G. Malbrán”, Buenos Aires C1282AFF, Argentina; nsanchezeluchans@gmail.com (N.S.E.);; 2 Servicio Antimicrobianos, Departamento de Bacteriología, Instituto Nacional de Enfermedades Infecciosas (INEI)—ANLIS “Dr. Carlos G. Malbrán”, Buenos Aires C1282AFF, Argentina; pgagetti@anlis.gob.ar (P.G.);

**Keywords:** *Streptococcus pneumoniae*, meningitis, pediatrics, vaccines, pneumococcal serotypes, antimicrobial resistance, invasive pneumococcal disease

## Abstract

The introduction of pneumococcal conjugate vaccination (PCV) into the Argentinian Childhood National Immunization Program in 2012 marked a significant milestone in public health. Our study aims to assess the impact of this intervention on pneumococcal meningitis cases, serotype distribution, and antimicrobial resistance among pediatric populations from 2013 to 2023. Specifically, we compared the early post-PCV period (2013–2014) to the late post-PCV period (2022–2023). A total of 333 pneumococcal isolates were analyzed between 2013 and 2023. Gold standard pneumococcal serotyping was performed to identify the serotypes associated with infection in children < 6 years in Argentina, and the agar dilution method was carried out to determine their profiles to antimicrobial agents. Our findings underscore the importance of PCV implementation, revealing notable shifts in pneumococcal epidemiology over the study period. The proportions of serotypes 1 (6.7% to 0.0%), 5 (5.6% to 0.0%), and 14 (7.8% to 1.8%) decreased, whereas the proportions of serotypes 10A (3.3% to 10.7%), 15B/C (2.2% to 10.7%), and 24B (0.0% to 8.9%) increased. The top five rated serotypes in the 2022–2023 period were serogroup 24 (21.4%), 10A (10.7%), 15B/C (10.7%), 23B (7.1%), and 12F (5.4%). Regarding antimicrobial resistance, we found that a total of 115/311 isolates (37%) were not suceptible to penicillin, and 2.9% were not suceptible to cefotaxime. Twenty-five percent of the isolates were microbial drug resistant, with resistance to penicillin, erythromycin, tetracycline/doxicycline, and/or cotrimoxazol. Among the PCV13 serotypes, 19A remained the most commonly associated with MDR. The non-PCV13 serotypes, particularly 24F, 24A, and 24B, were prevalent among MDR isolates. The observed trends demonstrate the need for the continued expansion of pneumococcal vaccination policies, including consideration for vaccines offering enhanced indirect protection, thereby extending benefits beyond the pediatric population to encompass adults as well. Such strategies are pivotal in reducing the burden of pneumococcal disease and safeguarding public health.

## 1. Introduction

Bacterial meningitis presents a significant public health challenge due to its potential for severe complications, including permanent sequelae [1,2]. While several bacteria can cause meningitis, *Streptococcus pneumoniae*, *Haemophilus influenzae*, and *Neisseria meningitidis* are the primary pathogens involved [3]. Incidence and mortality rates vary by region and age group [4], with pneumococcal meningitis being the predominant type globally, responsible for over 44,500 deaths and 2,720,000 years of life lost in 2019 [5].

Pediatric meningitis continues to pose a substantial public health challenge. As highlighted by Thigpen et al. [6] in their landmark study on bacterial meningitis, this disease can have devastating consequences for children, including hearing loss and even death. The potential for long-term complications, such as developmental delays and cognitive impairments, underscores the critical need for continued research and improved strategies for prevention, diagnosis, and treatment of this serious infection. Minimizing the burden of pediatric meningitis is essential for improving child health outcomes and ensuring a brighter future for affected children.

In Argentina, meningitis is a notifiable disease, requiring all suspected cases to be reported to the National Health Surveillance System (SNVS, its acronym in Spanish) [7], regardless of the underlying cause. The country also operates a laboratory-based surveillance system for bacterial invasive diseases. This system involves a network of public health and private hospitals that refer isolates to the National Reference Laboratories (NRL), INEI-ANLIS “Dr Carlos G. Malbrán”—Clinical Bacteriology and Antimicrobial Agent Divisions—for serotyping, genomic analysis, and antimicrobial susceptibility.

Conjugated pneumococcal vaccines play a pivotal role in diminishing the prevalence of invasive pneumococcal diseases and curbing the circulation of various serotypes [8]. Engineering to target specific polysaccharide capsules on the surface of *Streptococcus pneumoniae* enhances the immune response by conjugating polysaccharides with carrier proteins, thereby stimulating the production of protective antibodies. This immune response not only directly shields against targeted serotypes but also prompts a broader immune reaction, offering cross-protection against related serotypes [9]. Consequently, conjugated pneumococcal vaccines exhibit efficacy in thwarting invasive pneumococcal diseases like meningitis and bacteremia, as well as reducing pneumococcal carriage and transmission within vaccinated populations [8]. Given the dynamic nature of pneumococcal serotypes and the potential for serotype replacement post-vaccination, the use of conjugated pneumococcal vaccines proves invaluable in alleviating the burden of invasive pneumococcal diseases and managing serotype circulation, thereby contributing significantly to public health efforts aimed at curbing morbidity and mortality linked to pneumococcal infections [10].

The National Immunization Program of Argentina serves as a cornerstone in protecting public health by strategically addressing pathogens linked to bacterial meningitis [11]. In 2012, the program adopted the 13-valent pneumococcal vaccine (PCV13), effectively combatting pneumococcal meningitis and its related ailments. Such proactive measures underscore the commitment of Argentina to mitigating the burden of pneumococcal disease and safeguarding the well-being of its population.

Regarding this clinical manifestation, additionally, the World Health Organization (WHO) has committed to a roadmap with the ambitious aim of eradicating meningitis by 2030, highlighting the imperative of tackling its root causes [12]. Nevertheless, the onset of the COVID-19 pandemic has posed significant challenges to these plans, reshaping the landscape and affecting various aspects, including vaccine coverage. In 2023, the Argentine Ministry of Health released an epidemiological bulletin on meningitis, reflecting their ongoing efforts to monitor and control the disease’s incidence in the country. This update provided valuable insights into the current situation and trends [13]. Building on this, our study aimed to assess the impact of the PCV13 vaccine on pneumococcal meningitis by analyzing changes in serotype distribution and antimicrobial susceptibility over time. This research focused on children under 6 years old in Argentina, utilizing national disease surveillance data collected over an 11-year period.

## 2. Materials and Methods

In this study, we utilized a nationwide, population-based descriptive methodology complemented by a time-series analysis. We evaluated the distribution of serotypes causing meningitis in five periods from 2013 to 2023, drawing upon data from meningitis surveillance in Argentina: 2013–2014 (early post-PCV period), 2015–2016, 2017–2018, 2019–2021, and 2022–2023 (late post-PCV period)

To better understand the possible gap between SNVS and the NRL cases mentioned above, we conducted a comparison between the isolates submitted to the NRL and the cases reported to SNVS by year.

### 2.1. Collection of Bacterial Isolates

From January 2013 to December 2023, invasive isolates of *Streptococcus pneumoniae* from pediatric patients (<6 years old) were collected from 109 hospitals belonging to 24 jurisdictions as part of the National Surveillance Program.

Isolates and epidemiologic data were submitted to the NRL, INEI-ANLIS “Dr Carlos G. Malbrán”—Clinical Bacteriology and Antimicrobial Agent Divisions—for serotyping, susceptibility testing, and molecular analysis when required.

### 2.2. Isolate Analysis

#### 2.2.1. Serotype Detection Methodology

The serotypes or groups of all isolates were initially identified using the Quellung reaction with the commercial antisera (Staten Serum Institut, Copenhagen, Denmark) [14,15].

A minimal quantity of isolates that could not be serotyped via the Quellung reaction underwent confirmation through multiplex polymerase chain reaction (PCR) assays, as previously designed [16]. Isolates were designated as non-typeable (NT) only if both the Quellung reaction and the subsequent sequential multiplex PCR failed to classify them into one of the known serotypes, with confirmation via whole genome sequencing (WGS) validating this outcome.

#### 2.2.2. Establishing Serotype Categories

To delineate serotype categories, cases were organized based on the serotypes covered by various pneumococcal conjugate vaccines (PCVs). Specifically, the categorization included serotypes encompassed in PCV13 (1, 3, 4, 5, 6A, 6B, 7F, 9V, 14, 18C, 19A, 19F, and 23F), PCV15 (augmenting PCV13 with 22F and 33F) [17], PCV20 (extending PCV15 with 8, 10A, 11A, 12F, and 15B/C) [18], PCV24 (further including 2, 9N, 17F, and 20 to PCV20) [19,20]. Serotypes 15B and 15C were grouped as 15B/C due to their potential interchangeability arising from a slipped strand mispairing of a tandem thymine–adenine repeat [21]. Any serotypes not covered by the specified vaccines were classified as non-vaccine-type serotypes. We calculated the theoretical vaccine coverage by adding the proportion of each serotype included in each PCV for the first and last period.

#### 2.2.3. Vaccination Status of the Cases

The vaccination status of the cases was obtained from the mandatory epidemiological forms submitted alongside each isolate to the INEI-ANLIS “Dr Carlos G. Malbrán”—Clinical Bacteriology Division.

#### 2.2.4. Antimicrobial Susceptibility

Antimicrobial susceptibility testing was conducted using the agar dilution method to penicillin, amoxicillin, cefotaxime, meropenem, ceftaroline (from 2014), ceftobiprole (from 2017), erythromycin, clindamycin, tetracycline, doxycycline, chloramphenicol, cotrimoxazole, levofloxacin, rifampicin, and vancomycin following the guidelines outlined by the Clinical and Laboratory Standards Institute (CLSI) [22,23]. Ceftobiprole was interpreted according to EUCAST breakpoints [24]. Penicillin resistance was determined by a minimum inhibitory concentration (MIC) of ≥0.12 μg/mL based on meningitis breakpoints. For cefotaxime, MIC values of 1 μg/mL and ≥2 μg/mL were classified as intermediate and resistant, respectively. Quality control strains included *S. pneumoniae* ATCC 49619 and *Staphylococcus aureus* ATCC 29213.

Isolates displaying intermediate or full resistance were categorized as non-susceptible (NS), while multidrug resistance (MDR) was defined as non-susceptibility to three or more classes of antimicrobial agents [25].

### 2.3. Statistical Analysis

Statistical analyses were performed using R Studio (RStudio Team, versión 2024.04.1+748). To evaluate changes in the proportions of serotypes and antimicrobial non-susceptibility between the early post-PCV period (2013–2014) and the late post-PCV period (2022–2023), we conducted hypothesis testing using Fisher’s exact test, with a significance threshold set at a *p*-value of <0.05.

Several plots were employed for their effectiveness in illustrating the variation in isolates received across all months of multiple years. This visual tool enables us to observe the distribution of isolates per month for each year, facilitating comparison and identification of temporal patterns.

To examine the temporal distribution of the data over the years, we utilized appropriate statistical methods and conducted an ANOVA to assess the overall significance. The assumptions of normality and homogeneity of variances were evaluated using the Shapiro–Wilk and Bartlett tests on the residuals. Due to deviations of these assumptions, the Kruskal–Wallis test was employed as a non-parametric alternative to the ANOVA. Statistical significance was determined at an alpha level of 0.05.

### 2.4. Theoretical Vaccination Coverage

The theoretical vaccination coverage for PCV13, PCV15, PCV20, PCV21, PCV24, and PCV25 was estimated by calculating the percentage of serotypes targeted by each vaccine relative to all isolates. We assessed this vaccine coverage and explored its correlation with the serotypes identified in the Spn isolates. Our goal was to connect each identified serotype to the potential coverage offered by both existing and future vaccines available in Argentina.

## 3. Results

Between January 2013 and December 2023, a total of 1613 isolates from invasive pneumococcal disease in children under 6 years of age were summited to the National Reference Laboratory, INEI—ANLIS “Dr. Carlos G. Malbrán”. Of these isolates, 333—representing 20.6% of the total—were obtained from meningitis cases.

**Figure 1:**
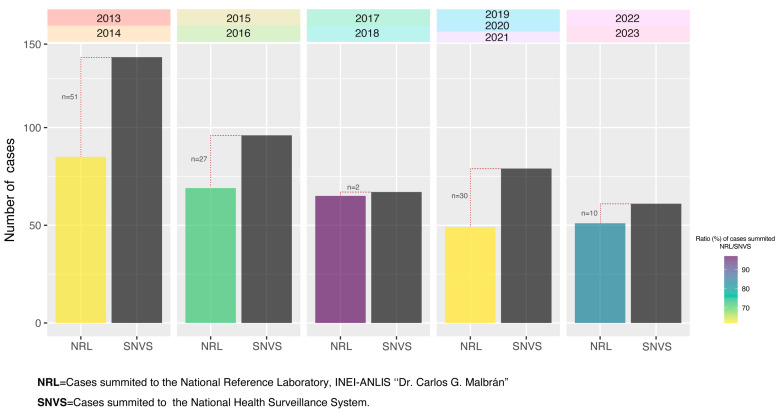
Comparation of cases summited to the NRL (National Reference Laboratory, INEI—ANLIS “Dr. Carlos G. Malbrán”) and to the SNVS. The plot represents isolates from individuals under 5 years old, as these are the data presented in SNVS. The number of cases reported to the SNVS was extracted from the Argentine National Epidemiological Bulletin (https://www.argentina.gob.ar/boletin-epidemiologico-nacional), last accessed on 11 September 2024.

Meningitis, as a notifiable disease, requires prompt reporting to the National Health Surveillance System (SNVS). Therefore, Figure 1 displays the correlation between the isolates submitted to the NRL and the cases reported to SNVS by period.

The average ratio of NRL cases to SNVS cases from 2013 to 2023 was 72.92%. However, this ratio increases to nearly 80% when excluding the pandemic years of 2020 and 2021.

**Figure 2:**
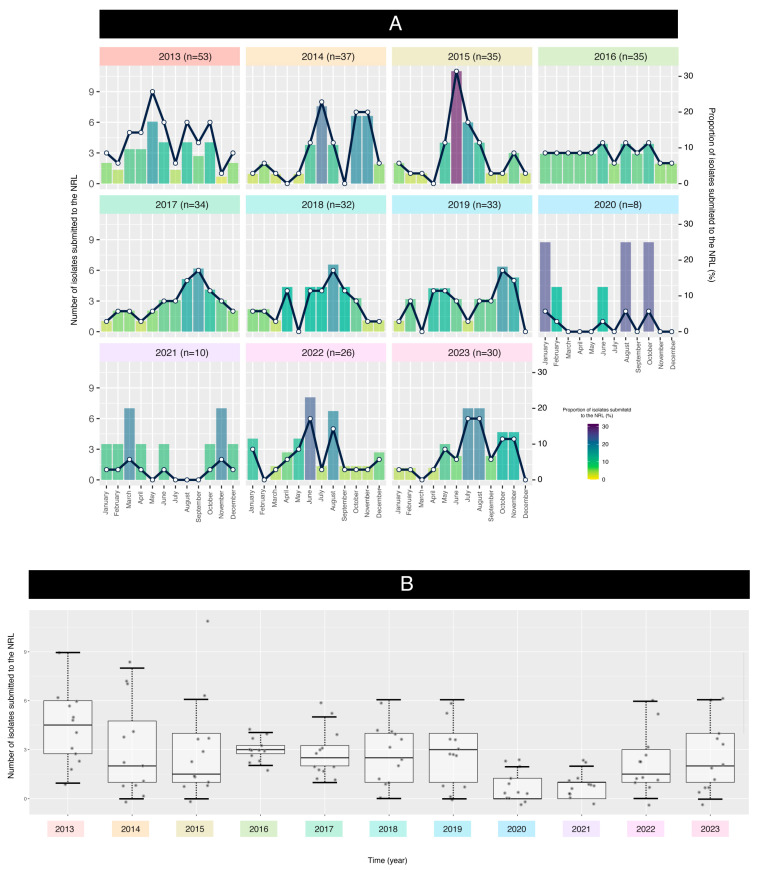
(**A**) Pneumococcal meningitis cases submitted to the National Reference Laboratory (NRL) during the study period. (**B**) Boxplot illustrating the distribution of isolates by year (the individual points of the boxplot represent the number of isolates submitted to the NRL for each month); the black boxes represent the interquartile range (IQR). The difference between years was analyzed using the Kruskal–Wallis test with multiple comparisons.

The plot and boxplot depicted in Figure 2A,B highlight the variations in isolate dates across different months over several years. Again, the submission of isolates was impacted by the SARS-CoV-2 pandemic, resulting in fluctuations in the proportion of isolates.

The data analysis aimed to assess the relationship between the year and the isolates submitted to the NRL. A linear regression model (LRM) was fitted to the data, treating years as categorical factors (i.e., not as a continuous variable) to assess temporal trends in the dataset. This approach allows for the identification of overall trends in the data without assuming a linear relationship between time and the variable of interest (number of isolates). The analysis revealed significant differences among the years (ANOVA *p*-value = 0.0007), indicating that the mean values varied significantly across the studied period. The model’s coefficient of determination (R^2^ = 0.217) shows that approximately 21.7% of the total variability is explained by the year-to-year differences. Figure 2B illustrates the significant differences in mean values across the studied years.

A residual analysis revealed violations of the ANOVA assumptions, including non-normality (Shapiro–Wilk test, *p* = 4.139 × 10^−5^) and heterogeneity of variances (Bartlett test, *p* = 2.638 × 10^−6^). Therefore, the Kruskal–Wallis test, a non-parametric alternative, was employed to assess differences between years. The Kruskal–Wallis test confirmed significant differences between years (Chi-squared = 35.211, *p* = 0.000115), indicating that the mean values across the study period were not equal. This finding highlights the presence of significant temporal variation in the data, likely influenced by the COVID-19 pandemic.

### 3.1. Serotype Distribution

**Figure 3:**
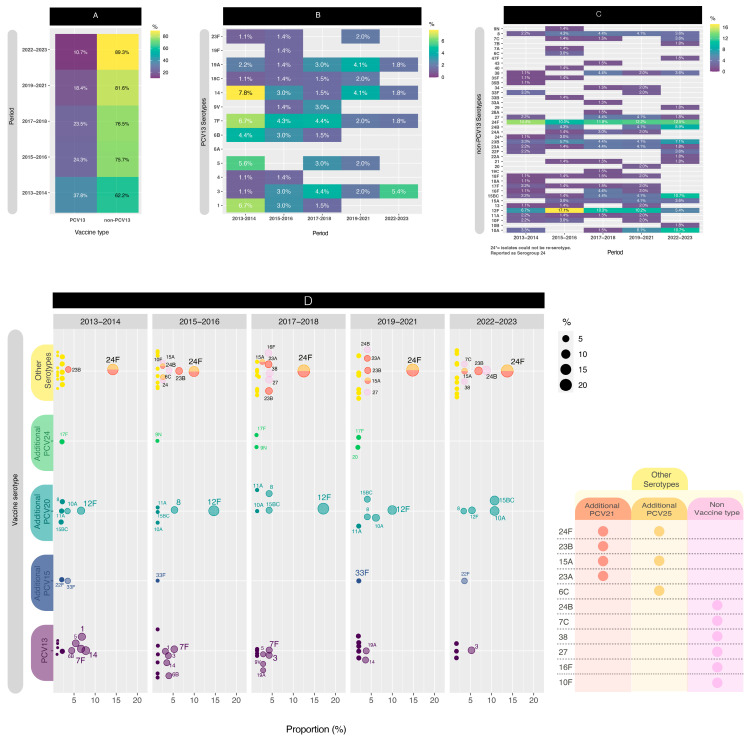
(**A**) Proportion of PCV13 isolates versus non-PCV13 isolates in the different periods studied. (**B**) PCV13 isolate distributions. (**C**) non-PCV13 isolate distributions. (**D**) Jitterplot of vaccine serotypes by year (points representing 3% or higher are labeled with 50% transparency). NVT = non-vaccine type.

For this segment of the analysis, we conducted a comparison across five distinct periods to enhance data visualization. Initially, we computed the distribution of PCV13 isolates versus non-PCV13 isolates (Figure 3A). Notably, beginning in the early post-PCV period (2013–2014), the proportion of PCV13 isolates exhibited a consistent decrease, from 37.8% to 10.3% (*p* < 0.05) in the late post-PCV period (2022–2023). The PCV13 serotypes, depicted in Figure 3B, revealed the presence of four serotypes (3, 7F, 14, and 19A) throughout all five comparison periods. When comparing the first period to the last period, significant decreases in PCV13 serotypes were observed for serotypes 1 (*p* < 0.05) and 5 (*p* < 0.05).

On the contrary, in Figure 3C, among the non-PCV13 serotypes, notable considerations included serogroup 24 and serotypes 15B/C, 23B, 12F, and 10A.

Although the values were relatively small, serotype 8 still persisted across all the periods evaluated.

Serotype 24F remained in high proportion in all periods, between 10.0% and 14.4%, while serotype 24B increased from 0.0% to 8.9% (*p* < 0.05). Serotype 23B also was present in all the periods, but its highest proportion (7.1%) was found in the late post-PCV period. Serotype 12F had a temporary increase from 6.7% in 2013–2014 to 17.1% in 2015–2016 (*p* < 0.05) and then decreased in the late post-PCV period to 5.4% (*p* < 0.05), with a value similar to the early-PCV period. On the other hand, serotypes 15B/C and 10A had a significant increase (*p* < 0.05) from 2015–2016 (1.4% and 0.0%, respectively) until the late post-PCV period (both 10.7%).

When comparing the early-PCV period to the late post-PCV period, significant decreases in PCV13 serotypes were observed for serotypes 1 (*p* < 0.05) and 5 (*p* < 0.05). Conversely, non-PCV13 serotypes that exhibited an increase in their proportion included 15B/C (*p* < 0.05) and 24B (*p* < 0.05).

In order to improve the visualization, we plotted the distribution of PCV13, PCV15, PCV20, and PCV24 serotypes by year (Figure 3D).

**Figure 4:**
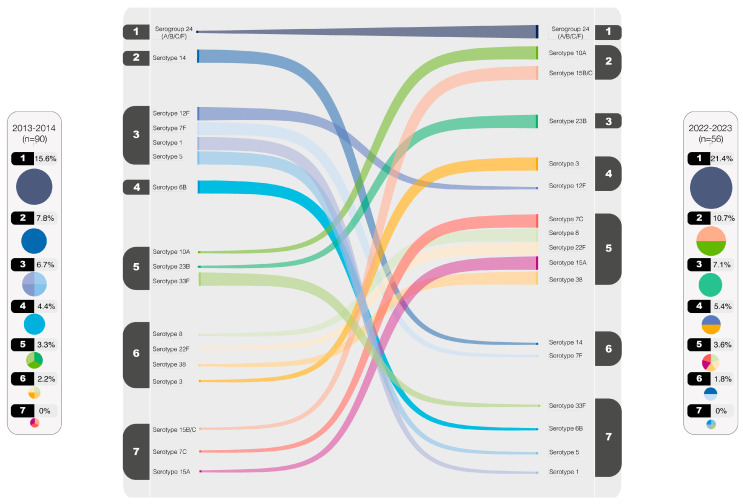
Comparative serotype distribution: 2013–2014 vs. 2022–2023 update.

In Figure 4, a comparison between the serotype rankings in 2013–2014 and 2022–2023 is illustrated. Serotypes with values equal to or greater than 2% were included in the plot. Notably, serogroup 24 has consistently maintained the position as the most prevalent throughout the observed periods. Conversely, certain serotypes, such as serotypes 1, 5, 6B, and 33F, have witnessed a significant decline in circulation. However, several other serotypes persisted among the top-rated, exemplified by serotype 3 emerging as the most predominant, with serotypes 7F and 14 following, albeit in lesser proportions.

The numbers from 1 to 7 represent the distribution of serotypes in descending order of prevalence, starting with the most frequent serotype (1) and progressing to the least frequent (7) based on their percentage.

**Figure 5:**
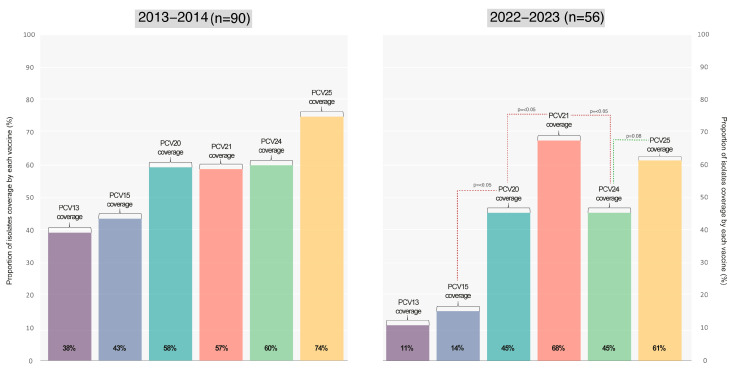
PCV theoretical coverage. Considering the period of 2022–2023, our analysis revealed no statistically significant differences between PCV13 vs. PCV15, PCV20 vs. PCV24, or PCV21 vs. PCV25. Of the currently available vaccines, PCV20 achieved the highest theoretical vaccination coverage of 45% in the most recent period.

The varying theoretical vaccine coverages provided by the current PCV and those anticipated from upcoming vaccines entering the market are displayed in Figure 5.

### 3.2. Assessment of Vaccination Status in Cases

**Figure 6:**
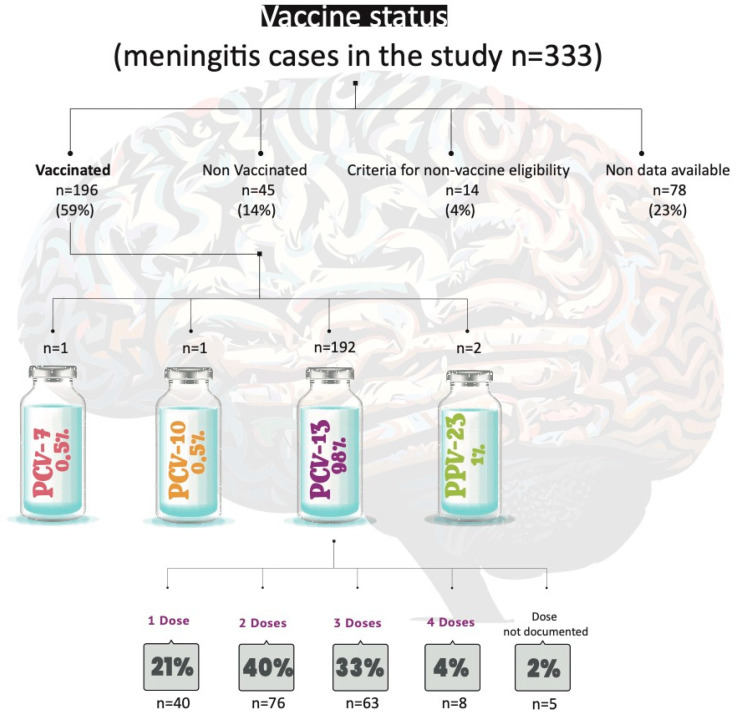
Vaccination status of the isolates studied. Criteria for non-vaccine eligibility indicate that the patients were not of the minimum age required for vaccination.

Figure 6 presents an overview of the vaccination status among the reported cases. This figure highlights the proportion of individuals who were vaccinated, partially vaccinated, or unvaccinated, providing insights into the relationship between vaccination coverage and the occurrence of cases within the studied population.

### 3.3. Antimicrobial Resistance Analysis

Antimicrobial susceptibility tests were performed on 311 isolates. Although not all of the antimicrobial agents evaluated are useful for the treatment of meningitis, susceptibility tests were performed as part of national surveillance.

Table 1 shows the antimicrobial resistance rates of the 311 pneumococcal isolates tested between 2013 and 2023. Considering the complete period of study, the resistance rates were as follows: penicillin, 37%; cefotaxime, 2.9%; meropenem, 3.9%; amoxicillin, 0.6%; cotrimoxazole, 46.6%; erythromycin, 28.0%; clindamycin, 21.9%; tetracycline and doxycycline, 32.2%. All of the isolates were susceptible to ceftaroline, ceftobiprole, rifampicin, vancomycin, levofloxacin, and chloramphenicol, and 78/311 isolates (25.1%) presented multidrug resistance overall. Fifty-seven isolates (18.3%) showed resistance to penicillin, erythromycin, tetracycline, and cotrimoxazole, and twenty isolates (6.4%) showed resistance to three of them.

If we focus on antibiotics useful for the treatment of meningitis, when comparing the first period (2013–2014) with the last one (2022–2023), significant changes were observed for penicillin but not for cefotaxime or meropenem. Regarding the other antibiotics tested, when comparing both periods, a significant increase was observed in resistance to erythromycin, tetracycline, and doxycycline (*p* < 0.05). MDR rates also showed a significant increase from 15.6% to 35.8% (*p* < 0.05).

Overall, serotypes 24A/B/C/F, 23B, 15B/C, 14, and 19A accounted for 75.6% of penicillin non-susceptible isolates, and serotypes 24A/B/C/F, 15B/C, 19A, 23A, and 6B accounted for 84% of multidrug-resistant isolates (Table 1). 

The distributions of the minimum inhibitory concentrations (MICs) for erythromycin, penicillin, cotrimoxazole, and tetracycline by serotype are available in the Supplementary Data (Figure S1).

## 4. Discussion

This study delved into a nationwide meningitis surveillance database to assess the impact of PCV13 on pneumococcal meningitis epidemiology within the paediatric population of Argentina over an 11-year period.

The analysis of isolates submitted to the National Reference Laboratory (NRL) provides a comprehensive overview of the national distribution of pneumococcal serotypes. Notably, the proportion of isolates submitted through the national surveillance network for invasive pneumococcal diseases, particularly meningitis isolates, showed a significant ratio between the SNVS report and isolates sent to the NRL. This ratio accounted for 73% of the total reported pneumococcal meningitis cases. However, when excluding 2020 and 2021—the pandemic years during which many laboratories and hospitals faced logistical challenges in submitting isolates to the NRL—this value rises to nearly 80%.

Following the introduction of childhood pneumococcal conjugate vaccines (PCVs), our study aligns with previous research [26,27,28,29] demonstrating a significant reduction in pneumococcal disease among children in Argentina. This impact is particularly evident in Supplementary Figure S2, which illustrates a continuous decline in the number of isolates submitted to the NRL within the 0–6-month age group, particularly for meningitis. Previous studies [30] have shown that meningitis in children under 1 year of age is associated with a high risk of mortality (10–15%) and neurological sequelae (30%). Furthermore, in the pre-PCV13 era in Argentina, meningitis and bacteremia were more prevalent than pneumonia during the first year of life, highlighting the critical importance of closely monitoring this vulnerable age group [31].

Also, this mirrors the findings of the PSERENADE project [32], which reported a decline in vaccine-targeted serotypes (≤26% across all ages) compared to the pre-vaccination era (≥70% in children). Argentina observed a similar shift, with the prevalence of PCV13 serotypes decreasing from approximately 87% in 2010 [13] to 10.7% in 2022–2023.

The Argentinean Ministry of Health reported that cases of pneumococcal meningitis in 2022 more than doubled compared to the previous biennium (2020–2021), which had the lowest case count of the past decade [13]. This low case count during 2020–2021 could be attributed to the COVID-19 pandemic, which led to lockdowns, social distancing, and personal protective measures. These measures resulted in a significant reduction in *S. pneumoniae* isolates, hospital admissions, and the incidence of invasive pneumococcal disease (IPD). While fluctuations in serotype circulation are evident, it remains uncertain whether these changes are directly linked to COVID-19 or reflect broader trends. The same report showed that in 2022, the incidence rate of pneumococcal meningitis in the global population was 0.3 per 100,000 individuals compared to pre-pandemic levels in 2019 (0.24/100,000 individuals). Additionally, the 2022 data highlight a potential vulnerability within the 2–4-year age group, aligning with findings from the LNR (see Supplementary Data, Figure S2). Here, incidence rates were slightly higher compared to the pre-pandemic levels in 2019 (0.36 vs. 0.45 per 100,000 individuals). This trend coincides with documented declines in pneumococcal vaccination coverage during the COVID-19 pandemic [33]. Vaccination coverage for both the two-dose primary series and the booster dose declined in 2020, reflecting a similar trend observed throughout the National Immunization Schedule. This decline further exacerbated the coverage gaps observed in previous years. One possibility is that this group received their primary series vaccinations closer to the onset of the pandemic, potentially leading to waning immunity if booster coverage remains low. Additionally, disruptions in routine healthcare access during lockdowns or pandemic-related anxieties could have also played a role in missed vaccinations.

As of this report date, the 2023 vaccination coverage for the primary series appeared similar to the 2021 levels, but coverage for the 12-month booster remained lower [34]. These coverage rates pose a challenge for achieving optimal disease control. Increased public health efforts are necessary to address vaccine hesitancy, improve access to routine immunizations, and ensure high booster coverage rates across all age groups. Continued monitoring of pneumococcal disease incidence and serotype distribution, alongside vaccination coverage data, is crucial to inform national immunization strategies and improve immunization coverage. This scenario can increase the number of individuals susceptible to infection, favoring the re-emergence of cases and the appearance of outbreaks caused by vaccine serotypes in the vulnerable population.

Among our findings, notable declines were observed in serotypes 1, 5, and 14, which are included in the PCV13 formulation, between the initial post-vaccination period (2013–2014) and the most recent period (2022–2023). However, it is noteworthy that serotypes 3, 7F, 14, and 19A remained present throughout 2019–2023, with a proportionate increase observed in serotype 3, although this was not statistically significant. This increase in serotype 3 requires continued monitoring in the coming years to determine if closer attention is necessary.

A comparison of serotypes between the PSERENADE project [32] and our study highlights the emergence of serotypes 24B, 7C, and 38 as notable “outliers” identified in our work but absent from the investigation by Garcia Quesada et al. Consistent with their findings, we also found that serotype 19A, a PCV13 serotype, was infrequent in our data (1.8%). Conversely, non-PCV13 serotypes such as 15B/C, 8, 12F, 10A, and 22F—associated with higher-valency PCV formulations currently under development—were prevalent in both studies (Table S1, Supplementary Data). Collectively, these serotypes accounted for 34% of pneumococcal meningitis cases during our most recent analysis period (2022–2023). Additionally, serotypes 24F and 23B, which are not included in the PCV15, PCV20, or PCV24 formulations, ranked among the most common serotypes in this timeframe.

A study by Lodi et al. from Italy, which examined only six cases of serotype 3 meningitis in children under 9 years old, suggested that serotype 3 may have varying impacts on different clinical presentations. In the post-PCV13 period, serotype 3 invasive pneumococcal disease (IPD) demonstrated an overall reduction in cases by 13% [35]. Particularly notable was the substantial reduction in cases of sepsis and meningitis, with a staggering 92% decrease among individuals born after the introduction of PCV13. While PCV13 appears to be effective against serotype 3 sepsis and meningitis in children, its efficacy against pneumonia is uncertain. However, De Wals et al. [36], in their review of immunologic and effectiveness data, delved into the indirect effects of PCV13 on serotype 3 disease. The study posited that while PCV13 may provide some level of protection in vaccinated children, this protection is likely to be less robust and possibly short-term compared to protection against other vaccine serotypes. This is in concordance with the results obtained in the present study, where serotype 3 consistently remained prevalent across all periods examined, averaging 3.2%.

The 13-valent conjugate pneumococcal vaccine (PCV-13) was incorporated into Argentina’s National Vaccination Schedule in 2012, utilizing a 2+1 scheme (administered at 2, 4, and 12–15 months of age) with the aim of mitigating the impact of pneumococcal pneumonia and IPD, thereby reducing morbidity and mortality associated with these conditions.

Subsequently, in 2017, a sequential vaccination strategy was initiated targeting individuals aged between 5 and 65 years with risk factors for IPD. This strategy involves the administration of both the 13-valent conjugate vaccine and the 23-valent polysaccharide vaccine and seeks to decrease the incidence, complications, sequelae, and mortality associated with pneumococcal pneumonia and IPD in these high-risk groups.

The current development and availability of the 20-valent conjugate vaccine (PCV20) presents an opportunity to reassess vaccination schedules, with the dual aim of enhancing the coverage of circulating serotypes and facilitating compliance with vaccination programs. In line with this, a new Technical Guidelines and Vaccination Manual was developed in 2024 [37]. This document outlines the national strategy for incorporating the 20-valent conjugate vaccine to replace the sequential PCV13-PCV23 schedule for individuals over 5 years of age with risk factors, as well as for those aged 65 and older.

In this manuscript, we conducted a thorough evaluation of the potential impact of both the current PCV (PCV13) and PCV15/PCV20, alongside promising vaccines (PCV21/PCV24/PCV25). The projected coverage outcomes of these vaccines align with our national epidemiological data, with PCV21 and PCV25 showcasing the highest anticipated coverage rates due to their serotype formulations.

While PCV21 and PCV25 offer expanded coverage by incorporating additional serotypes, it is essential to recognize that they exclude certain serotypes present in PCV13 such as serotypes 1, 4, 5, 6B, 9V, 14, 18C, 19F, and 23F for PCV21 and serotype 6A for PCV25. The implications of a future transition to these vaccines, once they are available, remain uncertain. Such a shift could lead to the resurgence of previously excluded serotypes, adding complexity to the epidemiological situation.

Regarding antimicrobial resistance, a significant increases in the proportion of resistance to penicillin, erythromycin, tetracycline, and doxicycline was observed throughout the study period, as well as in MDR. Non-PCV13 serotypes represented 88% of the MDR isolates, with 24F/A/B being the most prevalent.

As discussed above, in Argentina, the upcoming next-generation vaccines that would have the greatest impact on reducing antimicrobial resistance would be PCV21 and PCV25, which include serotype 24F. Unfortunately, neither includes serotypes 24A and 24B, which are also associated with MDR meningitis isolates.

This study has some limitations. The epidemiological data rely on forms completed by laboratories and hospitals, resulting in incomplete information about patients’ vaccination status (missing data account for 23% of the vaccination status information), chronic diseases, and risk factors. Additionally, outcome data were partially complete. We documented 35 deaths (10.6%) within the study population; however, this figure may not accurately represent the true mortality rate for this condition. According to the Ministry of Health report, the overall mortality rate for pneumococcal meningitis across all age groups was 9.9% in 2022 [12]. Of the 35 deaths documented in this work, 45% (16/35) were among vaccinated individuals, as reported in the epidemiological forms. Among these vaccinated patients, 18% (3/16) received one dose of PCV13, 50% (8/16) received two doses, 25% (4/16) received three doses, and 7% (1/16) had unspecified doses; however, based on their age, two doses would be expected. Notably, 31% (5/16) of the deaths among vaccinated patients were due to PCV13 serotypes, including two cases due to serotype 14 and three cases due to serotype 3. Intensified efforts to improve the collection of these epidemiological data by the NRL would enable the goal of enriching national surveillance analysis to be achieved in the near future.

Furthermore, not all meningitis cases reported to the SNVS correspond with the isolates submitted to the National Reference Laboratory (NRL). Although it is true that not all cases reported in SNVS were submitted to the NRL, this work shows that more than 70% of the cases registered in SNVS were studied in the NRL. One factor that could contribute to this discrepancy is that only hospitals participating in the National Surveillance Network typically submit isolates to the NRL. On the other hand, the increasing use of molecular methods for the detection of Spn in cases where the culture is negative could impact these observed differences. Undoubtedly, these methods improve the surveillance of invasive pneumococcal disease.

Another potential limitation of this study lies in the issue of multiple comparisons, which arises when testing numerous hypotheses using *p*-values. By nature, some comparisons may yield statistically significant results purely by chance, increasing the risk of false discoveries. Although this study focuses on key analyses relevant to the research questions, the potential for spurious findings cannot be entirely ruled out. Various approaches, such as the Bonferroni correction, are commonly employed to control for false discovery rates in multiple comparisons. However, in this study, we chose not to apply such corrections, prioritizing interpretative clarity in the context of exploratory analysis. Nevertheless, we acknowledge that there are different schools of thought regarding the handling of multiple comparisons, and some readers may prefer a more stringent approach to minimize false positives.

## 5. Conclusions

This study offers a comprehensive analysis of pneumococcal meningitis trends over 11 years, primarily following the introduction of PCV13 vaccination in Argentina. Our findings align with existing evidence, demonstrating a significant reduction in pneumococcal meningitis incidence among children targeted by the National Immunization Program’s PCV13 vaccination schedule. This reinforces the program’s effectiveness in preventing this serious childhood illness. We emphasize the relevance of monitoring the disease incidence, the serotype distribution, and its associated antimicrobial resistance to guide national pneumococcal vaccination policies and ensure optimal disease treatment and control.

## Figures and Tables

**Table 1:** Antimicrobial resistance rates to beta-lactam and non beta-lactam antibiotics of 311 pneumococcal isolates.

**Year**	**No. of Isolates (n)**	**Penicillin**		**Cefotaxime**		**Meropenem**		**Amoxicillin**		**Ceftaroline**		**Ceftobiprole**									
		**n**	**%**	**n**	**%**	**n**	**%**	**n**	**%**	**n**	**%**	**n**	**%**								
2013–2014	90	28	31.1	3	3.3	5	5.6	0	0	0	0	0	0								
2015–2016	61	23	37.7	2	3.3	3	4.9	0	0	0	0	0	0								
2017–2018	66	22	33.3	3	4.5	3	4.5	1	1.5	0	0	0	0								
2019–2021	41	16	39.0	0	0	0	0	0	0	0	0	0	0								
2022–2023	53	26	49.1	1	1.9	1	1.9	1	1.9	0	0	0	0								
Total 2013–2023	311	115	37.0	9	2.9	12	3.9	2	0.6	0	0	0	0								
**Year**	**No. of Isolates (n)**	**Vancomycin**		**Rifampicin**		**Chloramphenicol**		**Cotrimoxazole**		**Erythromycin**		**Clindamycin**		**Tetracycline**		**Doxycycline**		**Levofloxacin**		**MDR**	
		**n**	**%**	**n**	**%**	**n**	**%**	**n**	**%**	**n**	**%**	**n**	**%**	**n**	**%**	**n**	**%**	**n**	**%**	**n**	**%**
2013–2014	90	0	0	0	0	0	0	33	36.7	17	18.9	16	17.8	20	22.2	20	22.2	0	0	14	15.6
2015–2016	61	0	0	0	0	0	0	33	54.1	20	32.8	15	24.6	25	41.0	25	41.0	0	0	18	29.5
2017–2018	66	0	0	0	0	0	0	35	53.0	18	27.3	13	19.7	18	27.3	18	27.3	0	0	15	22.7
2019–2021	41	0	0	0	0	0	0	21	51.2	13	31.7	10	24.4	15	36.6	15	36.6	0	0	12	29.3
2022–2023	53	0	0	0	0	0	0	23	43.4	19	35.8	14	26.4	22	41.5	22	41.5	0	0	19	35.8
Total 2013–2023	311	0	0	0	0	0	0	145	46.6	87	28.0	68	21.9	100	32.2	100	32.2	0	0	78	25.1

## Data Availability

The original contributions presented in the study are included in the article/Supplementary Material, further inquiries can be directed to the corresponding author.

## Supplementary Materials

The following supporting information can be downloaded at: https://www.mdpi.com/article/10.3390/microorganisms13061301/s1, Figure S1. Distribution MICs for erythromycin, penicillin, cotrimoxazole and tetracycline by serotype. Figure S2. Distribution of cases submitted to the NRL in the period studied by group of age. Table S1. Serotype comparation between the PSERENADE project and this study.
