# Supplementary material for: Evolving Landscape of Paediatric Pneumococcal Meningitis in Argentina (2013–2023)
Source: Microorganisms. 2025 Jun 3;13(6):1301. doi: 10.3390/microorganisms13061301 (PMC12195527; doi:10.3390/microorganisms13061301)
Supplement: Supplementary file 1 [file microorganisms-13-01301-s001.zip › microorganisms-3417564-supplementary.pdf]

Supplementary Data

Figure S1. Distribution MICs for erythromycin, penicillin, cotrimoxazole and tetracycline by serotype.

Figure S2. Distribution of cases submitted to the NRL in the period studied by group of age.

Table S1. Serotype comparation between the PSERENADE project and this study.

Figure S1

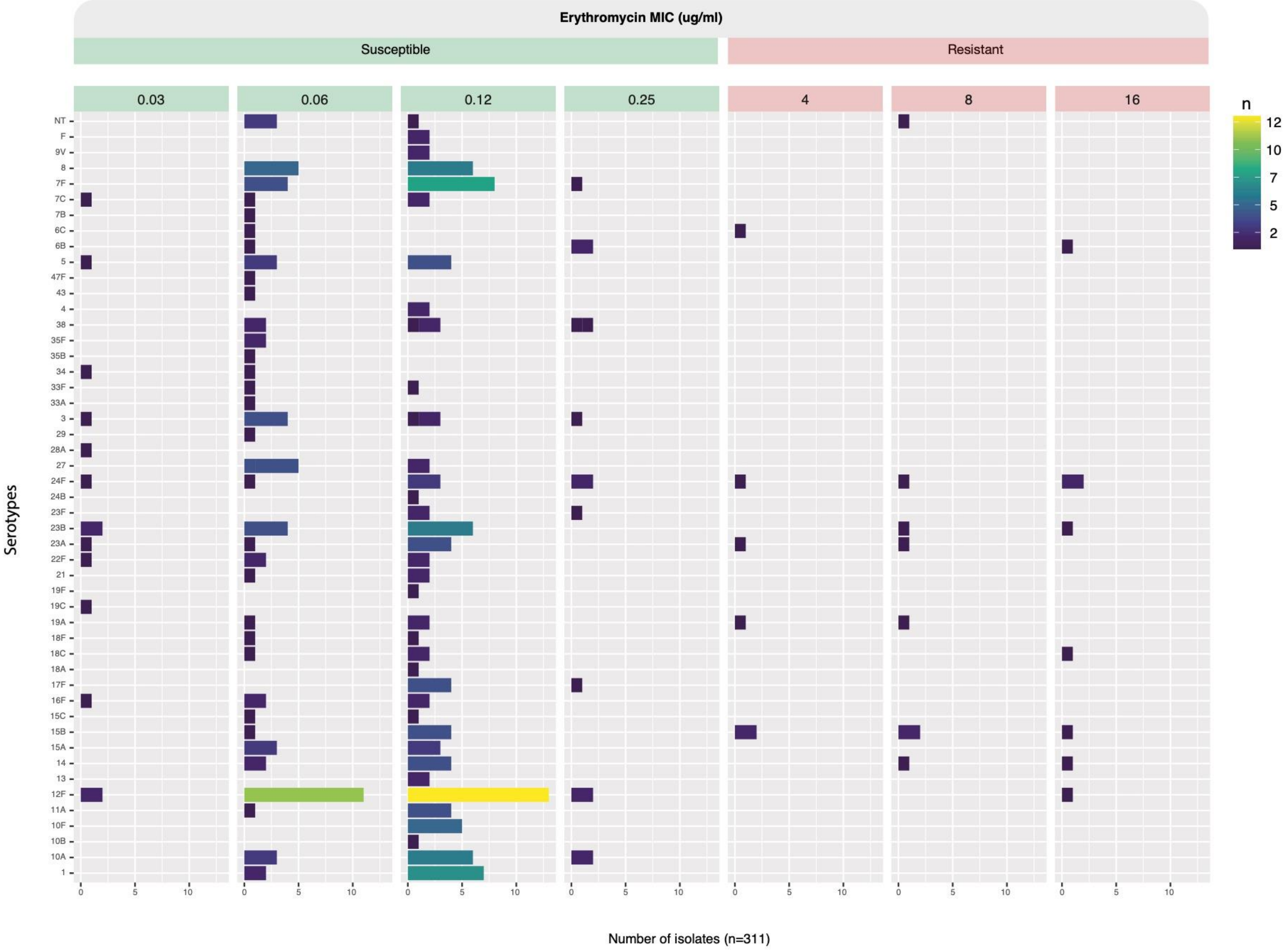

Erythromycin MIC (ug/ml)
Susceptible
Resistant
0.03
0.06
0.12
0.25
4
8
16
n
12
10
7
5
2
Serotypes
NT
F
9V
8
7F
7C
7B
6C
6B
5
47F
43
4
38
35F
35B
34
33F
33A
3
29
28A
27
24F
24B
23F
23B
23A
22F
21
19F
19C
19A
18F
18C
18A
17F
16F
15C
15B
15A
14
13
12F
11A
10F
10B
10A
1
0
5
10
Number of isolates (n=311)

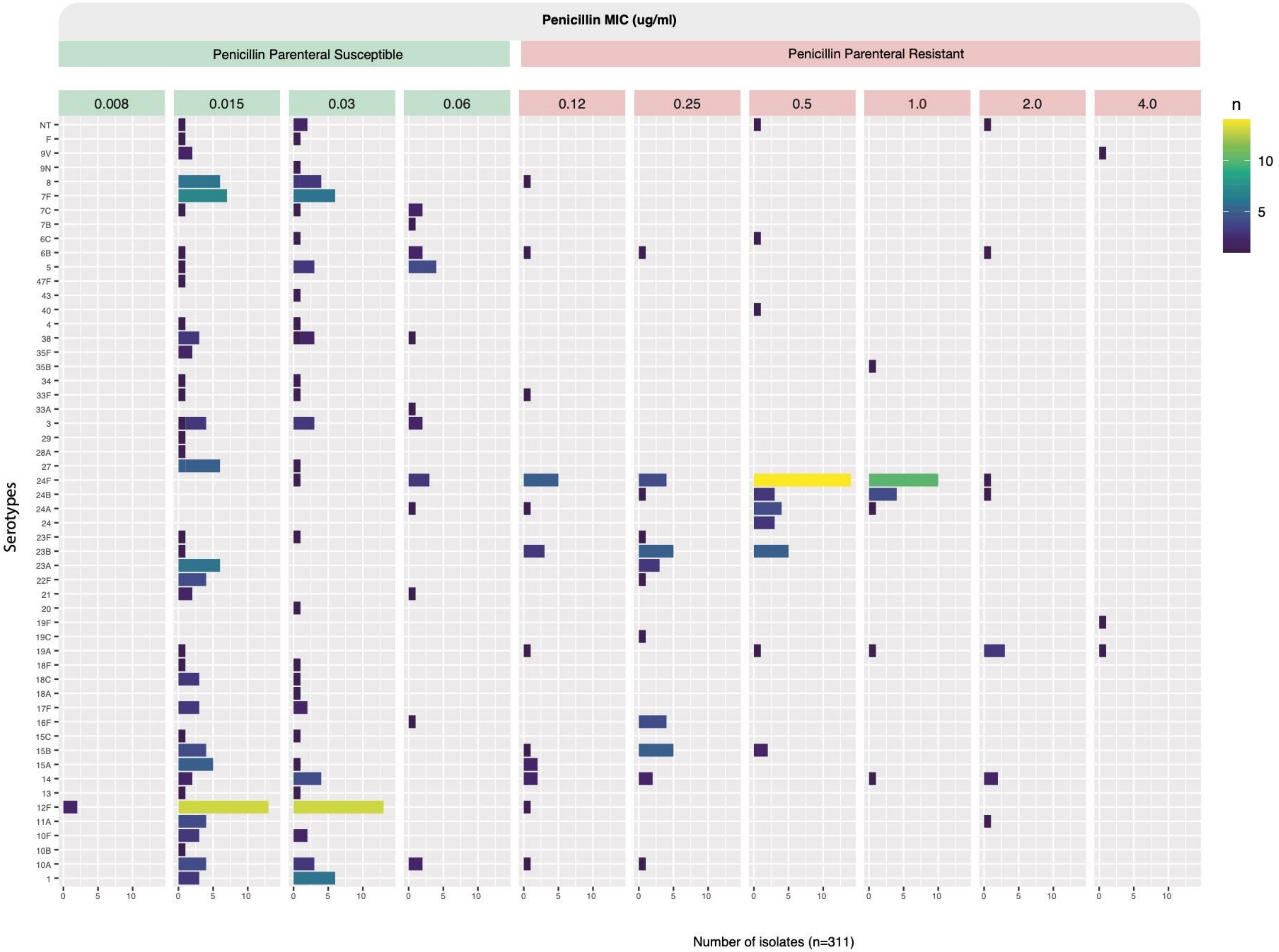

Penicillin MIC (ug/ml)
Penicillin Parenteral Susceptible
Penicillin Parenteral Resistant
0.008
0.015
0.03
0.06
0.12
0.25
0.5
1.0
2.0
4.0
n
10
5
Serotypes
NT
F
9V
9N
8
7F
7C
7B
6C
6B
5
47F
43
40
4
38
35F
35B
34
33F
33A
3
29
28A
27
24F
24B
24A
24
23F
23B
23A
22F
21
20
19F
19C
19A
18F
18C
18A
17F
16F
15C
15B
15A
14
13
12F
11A
10F
10B
10A
1
0 5 10
Number of isolates (n=311)

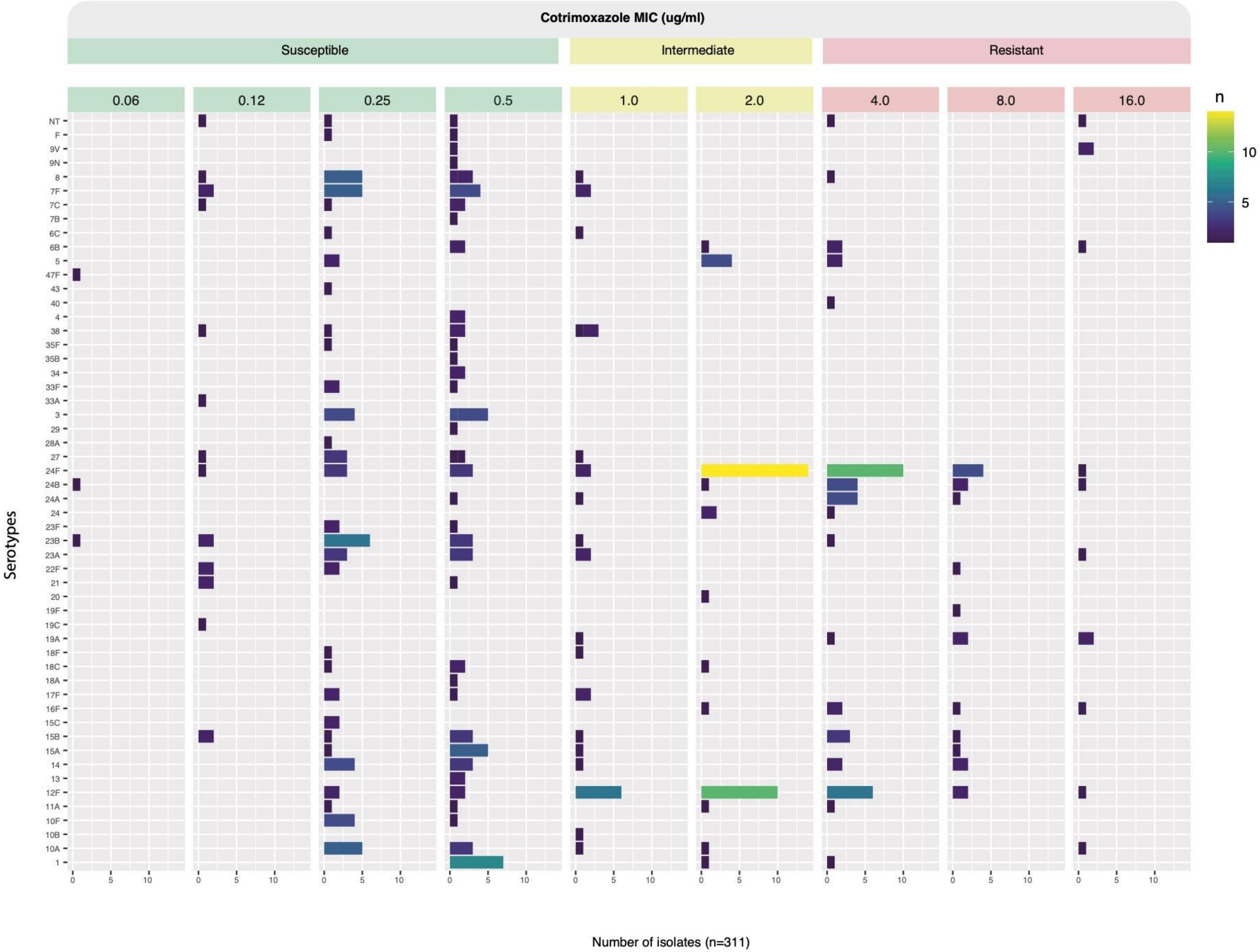

Cotrimoxazole MIC (ug/ml)
Susceptible
Intermediate
Resistant
0.06
0.12
0.25
0.5
1.0
2.0
4.0
8.0
16.0
n
10
5
Serotypes
NT
F
9V
9N
8
7F
7C
7B
6C
6B
5
47F
43
40
4
38
35F
35B
34
33F
33A
3
29
28A
27
24F
24B
24A
24
23F
23B
23A
22F
21
20
19F
19C
19A
18F
18C
18A
17F
16F
15C
15B
15A
14
13
12F
11A
10F
10B
10A
1
0
5
10
Number of isolates (n=311)

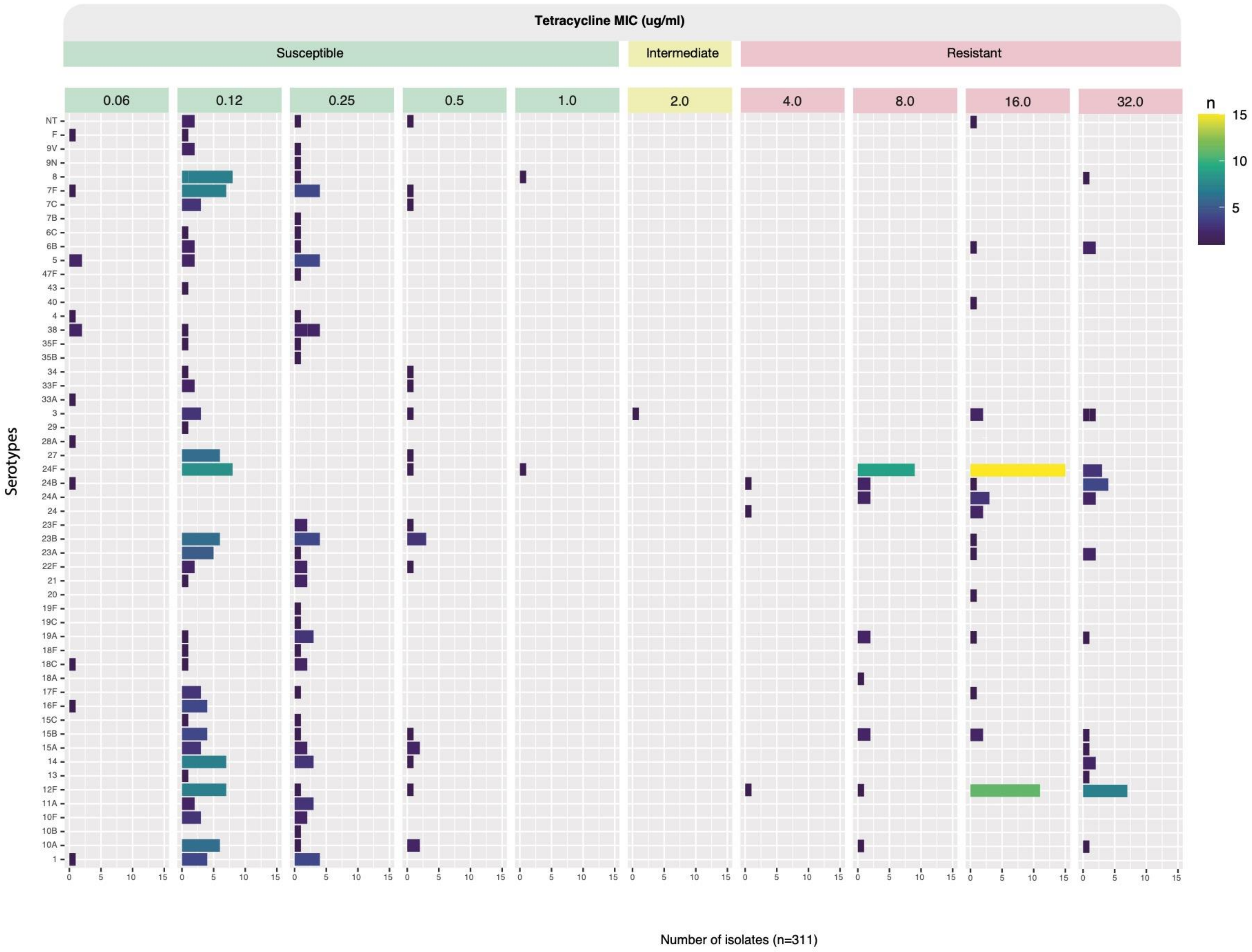

Tetracycline MIC (ug/ml)
Susceptible
Intermediate
Resistant
0.06
0.12
0.25
0.5
1.0
2.0
4.0
8.0
16.0
32.0
n
15
10
5
Serotypes
NT
F
9V
9N
8
7F
7C
7B
6C
6B
5
47F
43
40
4
38
35F
35B
34
33F
33A
3
29
28A
27
24F
24B
24A
24
23F
23B
23A
22F
21
20
19F
19C
19A
18F
18C
18A
17F
16F
15C
15B
15A
14
13
12F
11A
10F
10B
10A
1
0
5
10
15
Number of isolates (n=311)

Figure S2

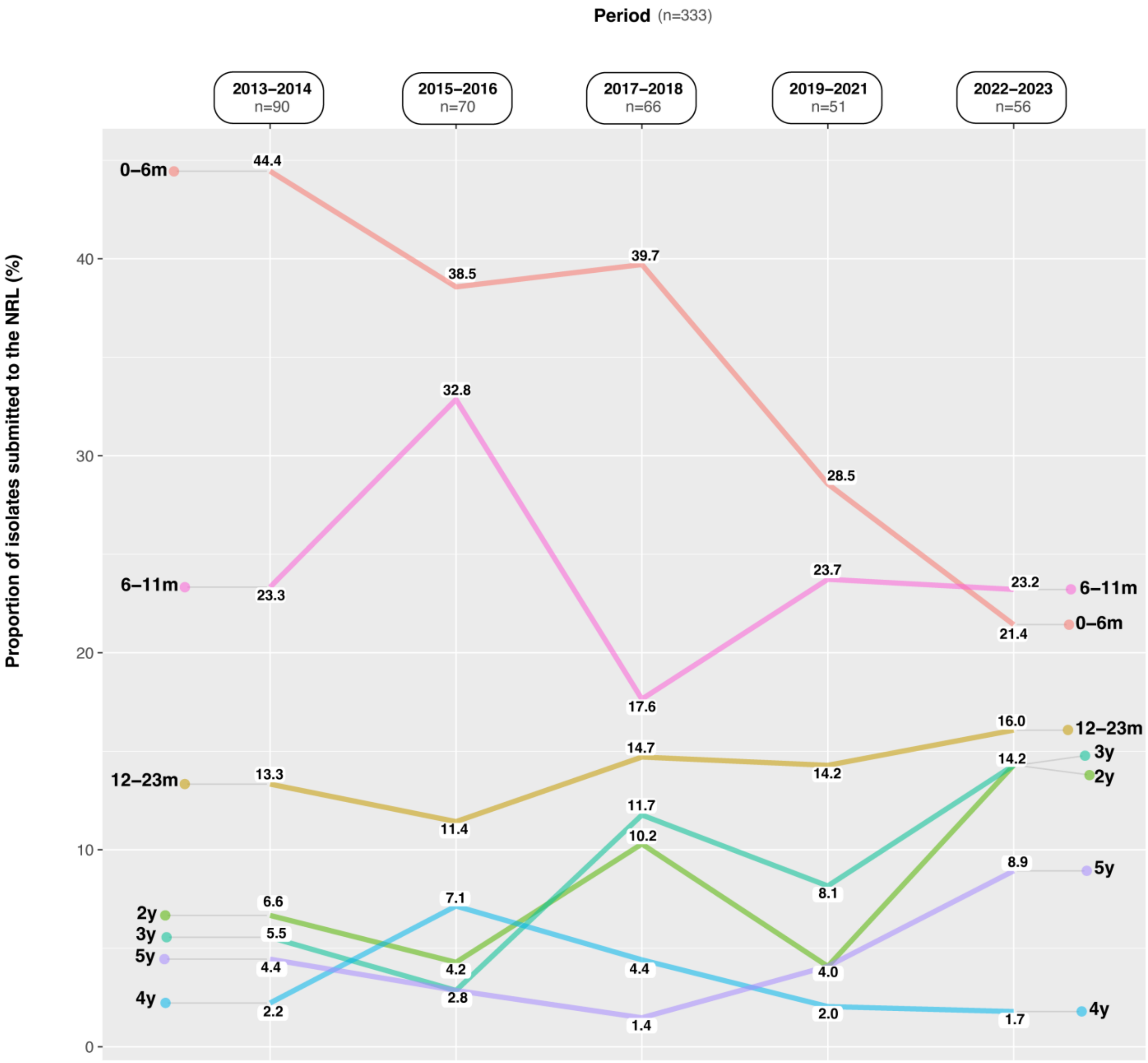

Figure S2. Distribution of cases submitted to the NRL in the period studied by age group.

Table S1. Serotype comparation between the PSERENADE project and this study.

	Proportion (%)	
Top rated serotypes	This study (<6y ,period 2022-2023)	PSERENADE Project (<5y)
24F	12.5	5.9
10A	10.7	7.1
15BC	10.7	11.5
24B	8.9	NA
23B	7.1	4.8
12F	5.4	4.6
3	5.4	4.0
8	3.6	7.1
22F	3.6	4.9
7C	3.6	NA
38	3.6	NA
15A	3.6	3.8

NA=not available
